# Safety and efficacy of a peripheral intravenous bolus of Licartin for the treatment of advanced hepatocellular carcinoma

**DOI:** 10.3892/etm.2013.1321

**Published:** 2013-09-30

**Authors:** DONG DAI, WENGUI XU, JIANJING LIU, LEI ZHU, XIANG ZHU, XIAOYING MA

**Affiliations:** Department of Molecular Imaging and Nuclear Medicine, Tianjin Medical University Cancer Institute and Hospital, National Clinical Research Center of Cancer, Key Laboratory of Cancer Prevention and Therapy, Tianjin 300060, P.R. China

**Keywords:** Licartin, hepatocellular carcinoma, radioimmunotherapy, safety

## Abstract

The aim of the present study was to examine the safety and efficacy of a peripheral intravenous bolus of Licartin for the treatment of advanced hepatocellular carcinoma (HCC), and to explore the clinical value of this treatment. Clinical data from Tianjin Medical University Cancer Institute and Hospital (Tianjin, China) were analyzed. Thirty-three patients (38 cases) with advanced HCC received an intravenous bolus of Licartin. The patients underwent routine blood examinations and liver, kidney and thyroid function tests 1 week prior to treatment and 1 and 3 months after treatment, and a long-term follow-up was performed. These data were collected before and after treatment was statistically analyzed and compared with that of previous studies regarding the safety of Licartin combined with transcatheter arterial chemoembolization for the treatment of HCC. During treatment, adverse reactions, including non-infectious fever, pain in the liver area, nausea and vomiting, occurred in a minority of patients. The adverse reactions were endured in the majority of cases and the symptoms were spontaneously relieved. Following treatment, 15 patients (39.47% of cases) demonstrated drug-related adverse reactions, including decreased white blood cell counts, platelet counts, hemoglobin levels and neutrophil counts, and increased levels of alanine aminotransferase, aspartate aminotransferase, serum direct bilirubin, creatinine and blood urea nitrogen, from high to low incidence. Electrocardiograms indicated no significant differences in thyroid function between patients before and after treatment, and showed stable vital signs. This study demonstrated that peripheral intravenous bolus administration of Licartin for radioimmunotherapy is safe and effective, is tolerated by the patient and may potentially become a routine treatment for HCC.

## Introduction

Hepatocellular carcinoma (HCC) is one of the ten most common malignant tumors worldwide, and its morbidity and mortality is increasing annually (1,2). There is a high incidence rate of HCC in China, with 292,966 new cases and 266,830 fatalities in 2008, which accounted for 40% of the total global incidence. Therefore, prevention and treatment methods for patients with HCC are required.

Treatment with radiopharmaceuticals, including ^131^I-lipiodol and microspheres, may be conducted by injection into the hepatic artery. The selective retention of the radionuclide in the liver tumor spares the normal tissues, which receive below the tolerated dose of internal radiation therapy (IRT) (3). Using HCC-associated antigens as a target, the specific binding of antigens and antibodies has been used to target the radionuclide to tumor lesions. This type of radioimmunotherapy (RAIT) has been increasingly studied for the treatment of cancer (4,5).

The therapeutic effects of Licartin (^131^I-lipiodol metuximab injection) are mainly implemented through the biological effects resulting from the ionizing radiation of ^131^I β-rays and the inhibition of signal transduction caused by the bound antibody fragments and targeting antigens (6,7). Specific and highly selective metuximab combines with liver cancer cell antigens (HAb18G/CD147) to result in the radionuclide ^131^I being targeted to and retained at the HCC tissues, while the vital tissues and organs have a low uptake. The high-dose concentrations of the radionuclide that are achieved irradiate the HCC and cause tumor cell death. The radiation doses in the whole body remain low and do not cause unrecoverable damage to other tissues and organs (8). HAb18G/CD147 is a newly discovered type of liver cancer-associated antigen. Studies have demonstrated that it is closely associated with metastasis and invasion (9–11); therefore, it may be used as an effective indicator for the early diagnosis of liver cancer and as an independent indicator of prognosis (12,13). Metuximab binds to the HAb18G/CD147 antigen on the surface of hepatoma cells and blocks HAb18G/CD147 antigen-induced signal transduction pathways, thereby inhibiting the metastasis and invasion of hepatoma cells and reducing liver cancer metastasis and recurrence. In pre-clinical studies, the control of tumor progression, extension of patient survival and improvement of quality of life were demonstrated (14).

In clinical studies, Licartin is locally administered via the hepatic artery and combined with transcatheter arterial chemoembolization (TACE). Due to efficient targeting, peripheral intravenous bolus administration of Licartin may be safer and more effective and convenient for radioimmunotherapy. However, no studies concerning the effects of peripheral intravenous bolus administration of Licartin exist in the literature. The present study analyzed a total of 33 patients (38 cases) with advanced liver cancer who attended the Tianjin Medical University Cancer Institute and Hospital (Tianjin, China) from October 2010 to July 2012 and received molecular imaging and Licartin radioimmunotherapy at the Department of Molecular Imaging and Nuclear Medicine. This study aimed to investigate the safety, efficacy and clinical applications of Licartin for the treatment of patients with HCC.

## Materials and methods

### General information

The study comprised 33 patients (38 cases) with advanced HCC who received Licartin (Chengdu Hoist Hitech Co. Ltd., Chengdu, China and the Fourth Military Medical University, Xi’an, China) in the Tianjin Medical University Cancer Institute and Hospital between October 2010 and July 2012. The study was conducted in accordance with the Declaration of Helsinki and with approval from the ethics committee of Tianjin Medical University Cancer Institute and Hospital. Written informed consent was obtained from all participants. Peripheral intravenous bolus injection was adopted for Licartin administration. The 38 cases comprised 29 who received Licartin treatment once, three who received Licartin twice and one who received Licartin three times. This study included 26 males and seven females (age range, 35–80 years; mean age, 46 years) with four, 15 and 14 cases in tumor node metastasis (TNM) stage II, III and IV, respectively. Patient information is shown in Table I.

### Pre-treatment preparation

All patients underwent a skin test of human anti-mouse antibody (HAMA) response, using one bottle of Metuximab injection (Chengdu Hoist Hitech Co. Ltd., Chengdu, China) dissolved in 1 ml saline. Dissolved solution (0.1 ml) was intradermally injected into the forearm. After 15 min the results were analyzed. If the flush diameter at injection point was >0.5 cm or the surrounding pseudopodia were observed, the skin test results were positive, and they were repeated until a negative result was achieved before treatment began.

To block and protect the thyroid gland, 0.5 ml compound iodine solution (Lugol’s solution; Department of Pharmaceutical Preparation in Tianjin Medical University General Hospital, Tianjin, China) was administered orally, three times a day for 10 days (from 3 days before administration of the bolus to 7 days afterwards). This was to avoid unnecessary radiation injury, which may be caused by the uptake of off-target radioactive ^131^I-lipiodol in the thyroid tissue.

### Drug delivery

Following the establishment of intravenous access or through peripheral veins, specified doses of ^131^I metuximab [27.75 MBq(0.75 mCi)/kg; maximum dose, ≤50 mCi] were injected slowly within 5–10 min. Tubes were immediately rinsed with 10 ml 0.9% normal saline to ensure full drug administration, while hepatoprotective symptomatic treatments were provided as appropriate.

### Treatment outcomes

The incidence and severity of nausea, vomiting, fever, pain and various adverse reactions were observed in all patients following treatment. Routine blood examinations and liver, kidney and thyroid function tests were performed 1 week prior to treatment and 1 and 3 months following treatment, and patients were regularly followed up. The preliminary assessment standard of the treatment effects were changes in α-fetoprotein (AFP) expression and from the imaging studies.

### Statistical analysis

Data are expressed as values or percentages. SPSS software, version 19.0 (SPSS, Inc., Chicago, IL, USA) was applied for statistical analysis and toxicity was tested by Wilcoxon rank-sum test. P<0.05 was considered to indicate a statistically significant difference.

## Results

### HAMA response

All patients underwent a skin test prior to metuximab injection; 15 min following injection, if the point flush diameter was >0.5 cm or pseudopods and blisters were observed, then this was regarded as a HAMA-positive reaction. Three months following the first Licartin treatment, four of the nine patients who were due to receive a secondary treatment did not receive further treatment owing to their HAMA reaction.

### Adverse reactions during treatment

Numerous patients experienced adverse reactions following the peripheral intravenous bolus administration of Licartin, including three cases (7.89%) with a non-infectious fever, four (10.53%) with burning sensation accompanied by liver area pain, two (5.26%) with nausea and one (2.63%) with vomiting (Table II). Compared with similar studies regarding the combination of Licartin administration and TACE, there was a lower incidence, fewer types and a reduced extent of adverse reactions in this group (specific classification is shown in Table II). In the present study, the majority of patients who tolerated the treatment demonstrated spontaneous remissions within 1 month.

### Electrocardiogram (ECG) results

An ECG was recorded 1 week before treatment and 1 and 3 months following treatment. No significant differences were identified from the ECG results following the treatment.

### Vital signs

Patient vital signs were observed 1 week before treatment and 1 and 3 months following treatment. There were no significant differences in all indicators before and after the treatment.

### Hematologic toxicity

Following treatment, 15 out of 38 cases (39.47%) experienced adverse reactions to Licartin including 11 cases (28.95%) with reduced numbers of white blood cells (WBCs), seven (18.42%) with decreased platelet (PLT) counts and seven (18.42%) with increased alanine aminotransferase (ALT) levels, six (15.79%) with increased aspartate aminotransferase (AST) levels, five (13.16%) with increased direct bilirubin (SDB) levels, four (10.53%) with reduced hemoglobin (Hgb) levels, four (10.53%) with neutral neutropenia, three (7.89%) with increased total bilirubin (STB) levels, one (2.63%) with increased creatinine (Cr) levels and one (2.63%) with increased blood urea nitrogen (BUN) levels.

Changes in hematological indices are shown in Table III. Following treatment, the levels of WBCs and PLTs decreased, while those of ALT and AST increased. These results were compared with data from phase II clinical trials for Licartin (15), and the probability of reductions of WBC and neutrophil levels was higher than that with local administration. However, the probabilities of reductions in PLT and Hgb levels and increases in ATL, AST, SDB, STB, CR and BUN levels was lower than that for local administration. Two independent sample t-tests indicated no significant difference in the probability of adverse reactions caused between the peripheral intravenous bolus administration of Licartin and local administration (P>0.05).

### Impact on thyroid function

Thyroid follicular cells have a strong ability to uptake and concentrate iodine, resulting in a concentration of iodide in the thyroid that is ≥20–25-fold greater than that in the plasma. Therefore, all patients in this study, before and following radioimmunotherapy, were orally treated with Lugol’s solution (0.5 ml three times a day for 10 days, 3 days before and 7 days after treatment) to block the thyroid tissue and avoid uptake of off-target radioactive ^131^I-lipiodol, which would result in unnecessary radiation injury.

In this study, 21.05% of patients indicated varying degrees of abnormal thyroid function. However, following treatment, a number of patients showed recovery of the abnormal thyroid, while certain patients who were euthyroid before treatment experienced thyroid dysfunction. The thyroid function changes before and after treatment are shown in Table IV. The vast majority of patients, before and following the peripheral intravenous bolus administration of Licartin, indicated no significant changes in thyroid function and the thyroid was successfully blocked and protected. One patient developed hypothyroidism ~3 months following the treatment. Analysis of the patient’s thyroid function showed thyroid stimulating hormone (TSH) levels were >100 mIU/l, and oral levothyroxine sodium alleviated the symptoms. According to the analysis, this patient demonstrated abnormal thyroid function before treatment (TSH=11.03 mIU/l) and did not receive a normal dose of Lugol’s solution, which may explain the ineffective blocking and protection of the thyroid.

### Clinical efficacy

In July 2012 (follow-up period, ≥3 months), in 33 patients, the clinical remission rate was 9.09% (three cases), the clinical efficiency was 21.21% (seven cases) and the clinical response (CR) rate was 60.60% (20 cases). The following are two specific example cases of the clinical efficacy of peripheral intravenous bolus administration.

**Figure 1:**
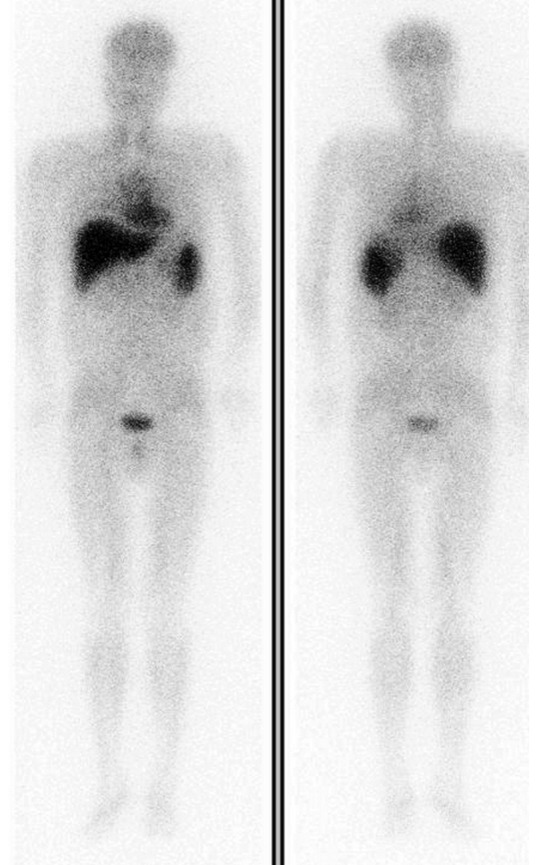
Case 1: Electrical capacitance tomography systemic imaging 8 days following the treatment showed aggregation of radiopharmaceuticals primarily in the liver and spleen, with amounts in the heart and bladder (normal physiological uptakes), and satisfactory closing and protection of the thyroid tissue. No apparent abnormal radioactive concentration areas were observed in other parts of the body.

Case one is a male patient (age, 42 years) with HCC who had undergone liver transplantation, and the pathological analysis indicated portal vein tumor thrombus; therefore, Licartin preventative treatment was administered. The patient received 30 mCi Licartin by intravenous injection on April 12, 2011. Eight days after treatment, an electrical capacitance tomography scan showed visible radioactivity in the surrounding liver area and bladder; however, the thyroid indicated no abnormal polyradioactivity, suggesting selective retention of the radionuclide (Fig. 1).

**Figure 2:**
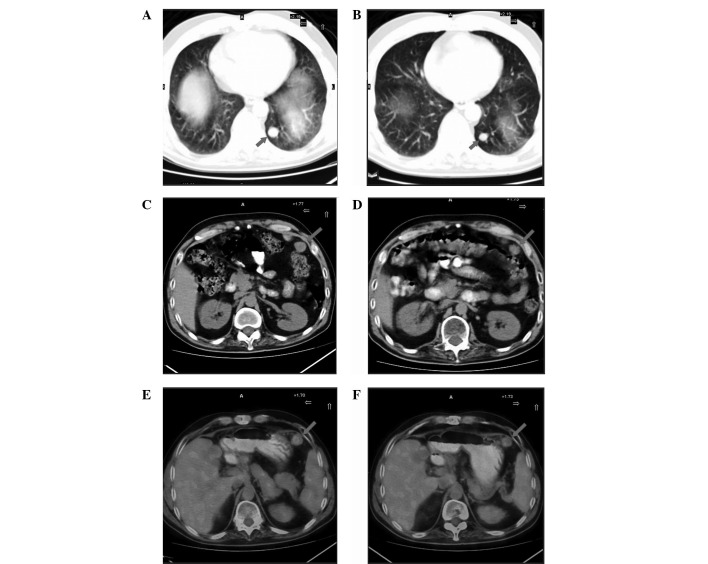
Case 2: (A and B) Pulmonary nodules. In the first month following Licartin treatment the volume of the lung metastatic lesions reduced (from 1.5×1.3 to 1.0×0.9 cm), the lesion density decreased and the computed tomography (CT) value reduced (from 39 to 5 Hu). (C and D) Mesenteric metastases. In the first month following Licartin treatment, a marginal reduction in the mesenteric volume (from 2.1×1.8 to 1.8×1.3 cm) was identified. (E and F) Side metastases of the stomach greater curvature. In the first month following Licartin treatment, the metastases of large curvature reduced (from 2.2×1.9 to 1.9×1.7 cm), the lesion center density was significantly lower, the CT value reduced (from 34 to 11 Hu), the degree of radioactive lesion aggregation was lower and the standardized uptake value reduced (from 3.2 to 1.8).

Case two was a male patient (age, 48 years) with TNM stage II and III who had undergone liver segment resection. Postoperative pathology of the left hepatic lobe demonstrated moderate and differentiated HCC. In the first year postoperative review, positron emission tomography-computed tomography (PET-CT) scanning identified a high recurrence probability of HCC in the left hepatic lobe, and basilar segment metastasis of the left lower lobe, peritoneal mesenteric multiple metastases and remnant liver metastasis near gallbladder fossa were observed. Following a clinical consultation, the patient received radioimmunotherapy with a 50 mCi intravenous bolus of Licartin on April 28, 2012. After 1 month, there were no evident symptoms and results of the routine blood test and liver and kidney function tests were normal. Additionally, the PET-CT scan showed that the HCC had improved (Fig. 2).

## Discussion

^131^I-labeled monoclonal antibodies are a biological treatment for cancer. ^131^I is targeted to the tumor site by an action-oriented antibody, while ^131^I-β-rays generate the biological effects. As the carrier, radionuclide-labeled antibodies concentrate in the tumor tissue and kill the tumor cells without destroying normal tissue. Compared with chemotherapy or radiotherapy, the toxicity of RAIT is low.

Pre-clinical studies have demonstrated that adverse reactions to Licartin are mainly transient blood toxicity and liver injury. In a phase II clinical study of Licartin (15) in 103 patients, there were 37 cases with drug-related adverse reactions (35.92%). The main adverse reactions were reductions in PLT (25.24%), WBC (18.45%) and Hgb (13.59%) levels, and an increases in ALT (21.36%), AST (21.36%), SDB (14.56%) and STB (8.74%) levels and proteinuria (8.74%). Wu *et al*(7) studied 110 patients with advanced liver cancer who received combined treatments of transhepatic arterial Licartin and TACE. The main adverse reactions identified were reductions in WBC and PLT counts; compared with the TACE treatment group, the incidence rates were significantly decreased (50.9 versus 21.2 and 42.7 versus 9.0%) and mainly stayed at I–II degree.

Owing to ischemia and hypoxia of the targeted liver tissue, as well as reperfusion injury and the side-effects of chemotherapy, varying degrees of liver dysfunction and hematological toxicity (16,17) have been observed with TACE treatment. In addition, ^131^I radiation injury accompanied by metuximab may mutually superimpose the two side-effects and aggravate the adverse reactions and liver damage from combination therapy. Therefore, studies have been conducted regarding combination therapy with TACE (18,19). Metuximab has effective targeting properties; it binds to HAb18G/CD147 on the surface of hepatoma cells to achieve maximum protection and avoid unnecessary liver damage and blood toxicity. In the present study, all patients received radioimmunotherapy intravenously by bolus administration of Licartin to assess the early adverse reactions and safety of the treatment for advanced HCC, and to explore its clinical value.

In the present study of 33 patients (38 cases) with liver cancer, 15 cases (39.47%) experienced possible drug-related adverse reactions, including 11 cases with a reduction in the number of WBCs (28.95%), seven with a reduction in the number of PLTs (18.42%), seven with increased ALT levels (18.42%), six with increased AST levels (15.79%), five with increased SDB levels (13.16%), four with a reduction in Hgb (10.53%) levels, four with neutropenia (10.53%), three with increased STB levels (7.89%), one with an increased CR (2.63%) and one with increased BUN levels (2.63%). Compared with intervention treatment, patients treated intravenously with Licartin indicated a reduced loss of liver function.

Due to strong iodine uptake and concentrating ability of thyroid follicular cells, a ≥20–25-fold greater concentration of iodide was observed in the thyroid compared with that in plasma; therefore, the changes in patient thyroid function were analyzed. Three months following the treatment, one patient experienced hypothyroidism; thyroid function tests showed that TSH levels were >100 mIU/l. However, following oral administration of levothyroxine sodium, this symptom alleviated. This may have been due to the insufficient blocking and protection of the thyroid, which were the result of abnormal thyroid function (TSH=11.03 mIU/l) and non-standard administration of Lugol’s solution before the treatment. This indicated that oral administration of Lugol’s solution before and after treatment may avoid unnecessary radiation injury and effectively protect thyroid function. Therefore, the volume of Lugol’s solution administered to patients should be regulated, supervised and patients must be reminded of the administration time.

The results of this study support the hypothesis that intravenous injection effectively reduces liver damage, which is conducive to the protection of liver function in patients with advanced HCC, increases the survival rate and significantly improves patient quality of life.

Short-term follow-ups were carried out for patients who received Licartin intravenous treatment. In July 2012 (follow-up, ≥3 months), the clinical remission rate of the 33 patients was 9.09% (three cases), the clinical efficiency was 21.21% (seven cases) and the CR rate was 60.60% (20 cases). According to the short-term follow-up results, Licartin demonstrated excellent targeting and inhibition of tumor progression, particularly in remote metastases, showing a unique advantage of Licartin radioimmunotherapy. The radionuclide ^131^I internal radiation effects occurred via binding to metastatic lesions, including those of the lungs, mesentery and stomach, by metuximab. Therefore, Licartin is a novel treatment for patients with HCC.

In conclusion, compared with intervention methods, intravenous bolus administration of Licartin demonstrated clear efficiency and mild adverse reactions. It effectively reduced liver injury and protected liver function in patients with advanced HCC, subsequently promoting patient survival. However, due to the relatively small number of cases in this study and the short-term follow-up of patients, a large-sample, multicenter clinical study is required for further analysis of the long-term effects of Licartin treatment.

## Figures and Tables

**Table I:** Patient information (n=33).

Variable	Value	Percentage (%)
Age (years)		
Median	46	
Range	35–80	
Gender (n)		
Male	26	78.79
Female	7	21.21
KPS ≥90 (n)	33	100.00
Child-Pugh stage (n)		
A	31	93.94
B	2	6.06
Hepatitis positive (n)		
B	32	96.97
C	1	3.03
TNM stage (n)		
II	4	12.12
III	15	45.45
IV	14	42.43
Tumor emboli (n)		
Yes	14	42.42
No	19	57.58
Abnormal rise of AFP (n)		
Yes	9	27.27
No	24	72.73
History of treatment (n)		
With radical surgery	24	72.73
Without radical surgery	9	27.27

KPS, Karnofsky performance status; TNM, classification of malignant tumors; AFP, α-fetoprotein.

**Table II:** Classification of adverse reactions in patients who received a peripheral intravenous bolus of Licartin.

	WHO acute and subacute toxicity grading of drugs, n (%)				
	
Adverse reactions	0	I	II	III	IV
Non-infectious fever	35 (92.11)	1 (2.63)	2 (5.26)	0	0
Liver area pain	34 (89.47)	4 (10.53)	0	0	0
Nausea	36 (94.74)	1 (2.63)	1 (2.63)	0	0
Vomiting	37 (97.37)	1 (2.63)	0	0	0

WHO, World Health Organization.

**Table III:** Classification of blood count, liver and renal function changes before and after treatment.

	1 week before treatment				1 month after treatment				3 months after treatment			
			
Indicators	0	I	II	III/IV	0	I	II	III/IV	0	I	II	III/IV
WBC	29	8	1	0	18	15	4	0	23	8	5	0
PLT	28	6	4	0	21	8	6	2	23	3	7	3
N	33	4	1	0	32	4	1	0	26	8	2	0
Hgb	33	3	2	0	33	4	2	0	30	6	0	0
ALT	30	8	0	0	30	6	1	0	28	6	1	1
AST	28	8	1	1	25	10	2	0	21	10	5	0
STB	32	4	2	0	33	3	0	1	27	8	0	1
SDB	33	4	1	0	32	4	0	1	34	2	0	0
Cr	31	3	0	0	28	4	0	0	35	1	0	0
BUN	36	2	0	0	31	5	1	0	31	5	0	0

Follow-up data are as follows: 38 cases before treatment, 37 cases (one patient succumbed within 1 month from acute upper gastrointestinal bleeding) 1 month following treatment, and 36 cases (a further case was lost) 3 months following treatment. Blood parameters [Center Common Toxicity Criteria (NCI-CTC) version 2.0] 1 week before treatment and 1 and 3 months following treatment were compared by Wilcoxon rank sum test to determine variation before and after the treatment. WBC, white blood cell; PLT, platelet; N, neutrophil; Hgb, hemoglobin; ALT, alanine aminotransferase; AST, aspartate aminotransferase; STB, total bilirubin; SDB, direct bilirubin; CR, creatinine; BUN, blood urea nitrogen.

**Table IV:** Changes in thyroid function before and after treatment.

Thyroidfunction	1 week before treatment, n (%)		1 month after treatment, n (%)		3 months after treatment, n (%)	
		
	Normal	Abnormal	Normal	Abnormal	Normal	Abnormal
T3	36 (94.74)	2 (5.26)	37 (100.00)	0	36 (10.00)	0
T4	37 (97.37)	1 (2.63)	37 (100.00)	0	34 (94.44)	2 (5.56)
TSH	33 (86.84)	5 (13.16)	34 (91.89)	3 (8.11)	32 (88.89)	4 (11.11)

TSH, thyroid stimulating hormone.
